# Surgical capacity assessment in the state of Amazonas using the surgical assessment tool. Cross-sectional study

**DOI:** 10.1590/0100-6991e-20223368-en

**Published:** 2022-09-09

**Authors:** JOSÉ EMERSON SOUZA, RODRIGO VAZ FERREIRAI, SAURABH SALUJA, JULIA AMUNDSON, ISABELLE CITRON, PAUL TRUCHE, LINA ROA, KATHRIN ZIMMERMAN, HILLARY E JENNY, ALEXIS N BOWDER, PEDRO HENRIQUE DA SILVA GOMES, JESSICA DE OLIVEIRA CORREIA, JOHN MEARA, NIVALDO ALONSO

**Affiliations:** 1 - University of the State of Amazonas, Department of Surgery - Manaus - AM - Brasil; 2 - Harvard Medical School, Program in Global Surgery and Social Change, Department of Global Health and Social Medicine - Boston - Massachusetts - Estados Unidos; 3 - Boston Children’s Hospital, Department of Plastic and Oral Surgery - Boston - Massachusetts - Estados Unidos; 4 - University of São Paulo, Department of Surgery - São Paulo - SP - Brasil

**Keywords:** Surgery, Safety, Quality Indicators, Health Care, Quality of Health Care, Health Care Quality, Access and Evaluation, Salas Cirúrgicas, Segurança, Indicadores de Qualidade em Assistência à Saúde, Indicadores Básicos de Saúde, Qualidade da Assistência à Saúde

## Abstract

**Objective:**

Brazil is a country with universal health coverage, yet access to surgery among remote rural populations remains understudied. This study assesses surgical care capacity among hospitals providing care for the rural populations in the Amazonas state of Brazil through in-depth facility assessments.

**Methods:**

a stratified randomized cross-sectional evaluation of hospitals that self-report providing surgical care in Amazonas was conducted from July 2016 to March 2017. The Surgical Assessment Tool (SAT) developed by the World Health Organization and the Program in Global Surgery and Social Change at Harvard Medical School was administered at remote hospitals, including a retrospective review of medical records and operative logbooks.

**Results:**

18 hospitals were surveyed. Three hospitals (16.6%) had no operating rooms and 12 (66%) had 1-2 operating rooms. 14 hospitals (77.8%) reported monitoring by pulse oximetry was always present and six hospitals (33%) never have a professional anesthesiologist available. Inhaled general anesthesia was available in 12 hospitals (66.7%), but 77.8% did not have any mechanical ventilation device. An average of 257 procedures per 100,000 were performed. 10 hospitals (55.6%) do not have a specific post-anesthesia care unit. For the regions covered by the 18 hospitals, with a population of 497,492 inhabitants, the average surgeon, anesthetist, obstetric workforce density was 6.4.

**Conclusion:**

populations living in rural areas in Brazil face significant disparities in access to surgical care, despite the presence of universal health coverage. Development of a state plan for the implementation of surgery is necessary to ensure access to surgical care for rural populations.

## RESUMO

**Objetivo:**

o Brasil é um país com cobertura universal de saúde, mas o acesso à cirurgia entre populações remotas permanece pouco estudado. Este estudo avalia a capacidade cirúrgica em hospitais que servem populações rurais no estado do Amazonas, Brasil, por meio de avaliações aprofundadas das instalações.

**Métodos:**

foi realizada avaliação estratificada randomizada transversal de hospitais que relataram prestar assistência cirúrgica de julho de 2016 a março de 2017. A Ferramenta de Avaliação Cirúrgica desenvolvida pela Organização Mundial da Saúde e o Programa de Cirurgia Global e Mudança Social da Harvard Medical School foi administrada em hospitais remotos, incluindo uma revisão retrospectiva de registros médicos e livros cirúrgicos.

**Resultados:**

18 hospitais foram pesquisados. Três hospitais (16,6%) não tinham salas cirúrgicas e 12 (66%) tinham 1-2. 14 hospitais (77,8%) relataram que a oximetria de pulso estava “sempre presente” e seis hospitais (33%) nunca têm um anestesiologista disponível. A anestesia inalatória estava disponível em 12 hospitais (66,7%), 77,8% não possuíam dispositivo de ventilação mecânica. Em média, 257 procedimentos por 100.000 foram realizados. 10 hospitais (55,6%) não possuem unidade de recuperação anestésica. Para as regiões de abrangência dos 18 hospitais, com população de 497.492 habitantes, a densidade média de força de trabalho cirúrgica, anestesista e obstétrica foi de 6,4.

**Conclusão:**

as populações que vivem em áreas rurais no Brasil enfrentam disparidades significativas no acesso à assistência cirúrgica, apesar da presença de cobertura universal de saúde. O desenvolvimento de um plano estadual de cirurgia é necessário para garantir acesso à assistência cirúrgica às populações rurais.

## INTRODUCTION

In 2015, the World Health Organization adopted resolution 68.15 declaring surgery an essential part of universal health coverage and critical for equitable improvements in global health and welfare^1^. This reinforced the need for access to safe surgical, anesthetic and obstetric care worldwide for the over five billion people do not have access^2^. A critical step towards improving access to surgical care, is evaluation of surgical system capacity in remote rural areas that present unique challenges with respect to surgical care delivery. 

Brazil is an upper-middle income country with an estimated population of 205 million. This population is spread across geographically diverse regions, but concentrated in the Midwest, South and Southeast^3^. This discrepancy is mirrored in the distribution of medical providers who are concentrated in major urban cities and state capitals^4^. Approximately 66% of physicians in Brazil practice in state capitals^5^. The Brazilian health system has three sectors. The public sector (Unified Health System - SUS) is financed federally and administered by the state governments. All Brazilian citizens have free access to hospitals and health services administered through the SUS system. The private sector is composed of hospitals that are not administered by the government in which services are paid directly by patient or covered through private insurance plans^6^. The Brazilian SUS system has been widely recognized as an example of successful healthcare reform in Latin America and have contributed to major improvements in access to health services across Brazil^7^ ^,^ ^8^. Despite these improvements, disparities in access to healthcare exist for rural populations in Brazil^9^ ^,^ ^10^. 

Health delivery in the state of Amazonas is complex due to expansive remote geography where remote cities often lack road access to the capital or other cities. A network of primary hospitals exists throughout the state to provide emergency and essential care, but all large tertiary referral centers are located in the capital city of Manaus. The delivery of surgical care at these remote primary hospitals has never been evaluated. 

This study aims to provide a cross-sectional assessment of surgical care capacity among hospitals serving remote populations in the state of Amazonas utilizing a previously validated surgical assessment tool. 

## METHODOLOGY

### Study Type and Sample Identification

This study was a prospective, randomized, cross-sectional, epidemiological study of hospitals across the state of Amazonas, Brazil conducted from July 2016 to March 2017. 

Data from the Department of Informatics of the Unified Health System (DATASUS) was reviewed to identify hospitals in the state of Amazonas that reported performing surgical procedures. Hospitals were included in this study if they reported performing any surgical procedure in 2015 in DATASUS. Any hospital that did not report a single surgical procedure in 2015 and all hospitals located in the capital city of Manaus were excluded as we chose to focus only on capacity among rural regions within the state of Amazonas and not the capital. 

**Figure 1:**
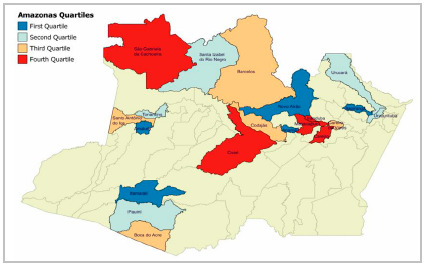
Distribution of randomly selected cities by quartile.

Fifty three hospitals met inclusion criteria for this study and were stratified into quartiles based on their catchment population. The first quartile included hospitals from 14 municipalities, and the remaining quartiles each included hospitals from 13 municipalities. Five hospitals were randomly selected from each quartile. A total of 20 hospitals were selected (Figure 1). 

#### Outcome Measures

The Surgical Assessment Tool (SAT)^11^ was used to perform an in-depth facility assessments. The SAT is a modified version of the Hospital Assessment Tool developed by the World Health Organization (WHO) as part of the Integrated Management for Emergency and Essential Surgical Care (IMEESC) toolkit. The initial SAT tool was piloted in Cape Verde, Ethiopia and India. The tool was then adjusted and validated by 18 Experts (Delphi consensus) and piloted at the Regional Referral Hospital for Mbarara in western Uganda^12^. The SAT can be used to evaluate the strength of a surgical system within 5 domains: infrastructure, service delivery, workforce, information management, and financing. Of note, we did not perform the financing portion as care provided at all hospitals surveyed was covered by the national health system with no point of care costs for patients. 

The majority of survey questions are assessed on a six-point Likert scale (always (100% of the time), almost always (76-99% of the time), most of the time (51-75% of the time), sometimes (26-50% of the time), rarely (1-25% of the time), and never (0% of the time). Questions also quantify the number of surgical procedures, providers and resources.

### Data collection

The SAT was used to conduct interviews of hospital administrators and frontline providers. In addition, a 30-day retrospective review of medical records was completed. All respondents were adults over 18 years of age who gave informed consent. All interviews took place in the respondent’s own hospital and were conducted in Portuguese by one of the authors The following personnel were interviewed: the hospital administrator, surgeons, anesthesiologists, obstetricians, and surgical unit nurses. Data collection was performed through on-site visits to each hospital by site surveyors from the University of Amazonas (JE, RVF, PHG, JC). 

We assessed the internal validity of the tool by performing a revisit of two randomly selected hospitals 30 days later and comparing the data collected for each hospital. Two hospitals (Manacapuru and Iranduba) were randomly selected and revisited 30 days after the initial data collection period in order to assess internal validity of the study. The facility assessment was conducted. Second time and data between the two assessments were compared in order to determine if there were significant changes in reported data at two different time intervals.

### Ethical considerations

This project was conducted in partnership with Boston Children’s Hospital, University of São Paulo (USP) and University of State of Amazonas (UEA). Institutional Review Board approval was obtained from the UEA (Approval number 1,5225,514) and the USP (Approval number 1,904,101). This study was determined to be IRB exempt by Boston Children’s Hospital

### Statistical analysis

Descriptive statistics were performed. Comparisons were made using Fisher’s exact, chi squared, and Wilcoxon rank-sum as appropriate. Analysis was performed using the IBM SPSS statistical program Statistics Version 21. 

## RESULTS

Eighteen of the 20 hospitals selected underwent facility assessment. Two hospitals (Itamarati and Barcelos) were selected for inclusion, but only accessible by private helicopter and unable to undergo assessment. The remaining hospitals were accessible by road or boat. All 18 were noted to be the only public hospital serving the population of that city and surrounding municipality and no private hospitals were identified in any of the municipalities served by these hospitals. The 18 hospitals visited represented municipalities a total population of 497,492 inhabitants, or 25.71% of the Amazonas population (Table 1). 

**Table 1:** Population of cities.

Municipality	Population
Amaturá	9.467
Anamã	10.214
Boca do Acre	30.632
Brown Careiro	32.734
Careiro da Várzea	23.930
Coari	75.965
Codajás	23.206
Iranduba	40.781
Itapiranga	82.110
Manacapuru	85.141
New Airão	14.723
Pauini	18.166
Santa Isabel do Rio Negro	18.146
Santo Antônio do Iça	24.481
São Gabriel da Cachoeira	37.896
Tonantins	17.079
Urucará	17.094
Urucurituba	17.837

### Infrastructure

**Figure 2:**
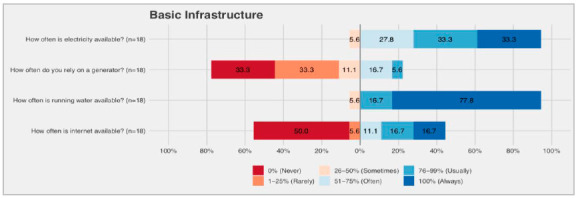
Basic infrastructure.

Regarding availability of electricity, six (33.3%) hospitals reported ‘almost always’ having electricity and six (33.3%) reported ‘always’ having electricity. One hospital (5.6%) reported only “sometimes” having electricity, and one reported not having access to a generator. Five (27.8%) hospitals never needed to use a generator and six (33.3%) rarely used it. The water supply was always available in 14 (77.8%) hospitals and ‘almost always’ in three (16.7%). Internet access was severely limited in municipalities in the interior of the state, and ‘always present’ in only three (16.7%) hospitals and ‘never present’ in nine (50%). All hospitals reported having oxygen supplied by cylinders except one hospital which supplied oxygen through plumbing (Figure 2). 

**Figure 3:**
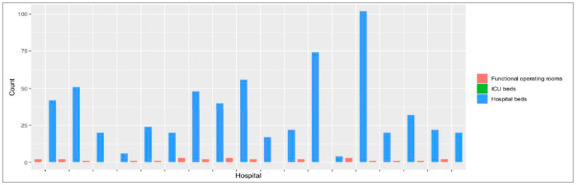
Characteristics of operating rooms and number of beds.

A combined total of 620 beds were available among the 18 hospitals surveyed with an average of 34 beds per hospital (Range 4-102). None of the hospitals studied had an intensive care unit (ICU). The majority of hospitals had 1-2 operating rooms (Figure 3). In three hospitals (16.7%), no operating rooms were found. 

**Figure 4:**
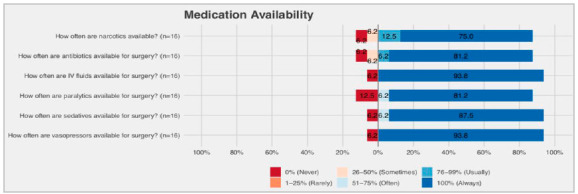
Characteristics of medication availability.

Over half of the hospitals (55.6%) did not have a specific post-anesthesia care unit (PACU). Only one (5.6%) hospital had four beds for post-anesthetic care and three (16.7%) hospitals had two beds. Six hospitals (33.3%) never had a professional anesthesiologist available, and only four (22.2%) report universal availability. In 14 hospitals (77.8%), there was no nurse in the PACU. Sedatives, paralytic and antibiotics were ‘always available’ in most hospitals (Figure 4).

### Service Delivery

There were 1281 procedures performed at the 18 hospitals over six months. This included 603 caesarean sections, 49 laparotomies, 12 open fractures, and 617 minor surgeries (e.g. excision of lipomas or sebaceous cysts, circumcision). No facility used the WHO surgical safety checklist. All hospitals reported a perioperative mortality rate of zero. Pulse oximetry monitoring was ‘always present’ in 13 (77.8%) of the hospitals. Inhaled general anesthesia was available in 12 (66.7%) hospitals, but the vast majority of hospitals did not have mechanical ventilation devices (14, 77.8%). Access to local and spinal anesthesia was “always” present in 14 hospitals (77.8%) of hospitals.

### Surgical Workforce

**Figure 5:**
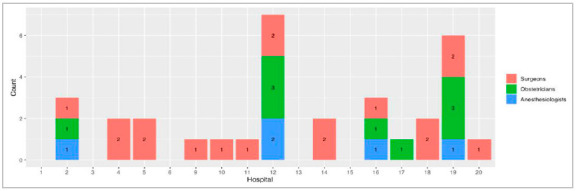
Providers available one day per week.

**Figure 6:**
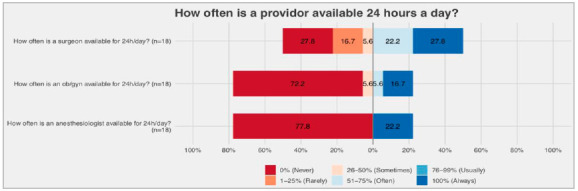
Providers available 24 hours per day.

Thirty-two surgeons, anesthesiologists and obstetricians (SAO), providers were present at the hospital’s headquarters at least once a week, resulting in a surgical work force of 6.4 SAO for every 100,000 inhabitants. However, these providers were not available 24/7. Eighteen (56%) were general surgeons, nine were gynecologists/obstetricians (28%) and five were anesthesiologists. Due to the lack of anesthesiologists, some surgeons performed anesthesia for their own procedures. Additionally, as some providers were not available 24/7, generalist medical professionals holding a bachelor’s degree in medicine sometimes performed surgical procedures when no surgeon was available. Providers who were available at least 1 day per week per hospital are shown in Figure 5. Providers available 24 hours per day are shown in Figure 6.

### Information Management

Each facility had a designated person responsible for storing and organizing medical data. 14 (77.8%) still used paper records, and 6 (33.3%) always collected postoperative data such as surgical site infections, complications, and mortality. One facility (5.6%) uploaded the data weekly to the Ministry of Health, while the others uploaded their data monthly.

## DISCUSSION

Despite the presence of universal health care, significant gaps in access to surgical care exist for the rural populations we studied due to significant limitations in surgical capacity and workforce in Amazonas. Although remote hospitals across the region provide emergency and essential surgery, our results show that few hospitals are fully equipped to perform operations and are significantly limited by a lack of surgical, obstetric and anesthesia workforce. 

The lack of SAO workforce at rural hospitals is a major barrier to 24/7 access to safe, high quality surgery. The limited number of SAO workforce reflect the physician distribution where of the 4,844 doctors in Amazonas, 93% work only in the capital. Our study reveals that this means only 23 surgeons, 16 anesthesiologists and 22 obstetricians cover the remaining 18 municipalities in the state of Amazonas4. The SAO density of hospitals sampled in our study is 6.4 per 100,000 which is well below the recommended minimum target of 20-40 SAO outline in the Lancet Commission for Global Surgery and less than both Brazils national SAO density of 34.7 and the state of Amazonas SAO density of 18.42^2^ ^,^ ^13^. Much of the SAO workforce that does work in rural hospitals, provide services only on a part time basis. In Brazil, more than 70% of doctors in the public sector also work in the private sector^14^. In rural areas, it is not possible for physicians to work in the private sector to supplement their income, and thus remote hospitals rely on physicians who work short 10-15 day periods. 

For the state of Amazonas, we found low volumes of surgical procedures performed at surgically capable rural hospitals. Among hospitals we studied, only 1281 surgeries were performed in 18 hospitals over six months. This equates to an average of 257 procedures per 100,000, which is much lower than the Lancet Commission on Global Surgery (LCoGS) indicator target of 5,000 operations per 100,000 people^2^. C-Sections, often performed under spinal anesthesia, were the most commonly performed procedure likely reflecting a lack of anesthesia providers to provide general anesthesia. The surgical volume we noted is in contrast the capital of Manaus where 84,795 surgical procedures were performed in the same time period, resulting in a volume of 4,037/100,000 people^13^. This demonstrates the state’s almost complete reliance on the capital for surgical care. 

With regard to basic infrastructure, our findings are better than those found in other Low and Middle Income Countries (LMICs) where district hospitals routinely face water, oxygen, and electricity shortages^15^ ^-^ ^17^. The primary infrastructure limitations result from limited capacity in terms of the number of operating rooms and the lack of intensive care units which limits capabilities to perform complex emergency operations^18^. 

Brazil provides universal health coverage (UHC) through federal mandate which aims to provide free, high quality care to all Brazilians regardless of ability to pay which has been recognized as a way to achieve the United Nations Sustainable Development Goals^19^. Rural populations in Brazil depend on the public system for surgical care as only 13% of the Amazonas population is covered by private health plans and 97.7% of this population resides in the capital of Manaus^20^. Despite the presence of UHC for rural populations in Brazil, there remains significant barriers to reach surgical care especially for emergent patients who do not have the luxury of being flown to the capital. 

Optimizing the delivery of surgical care for the state of Amazonas will require both investment in infrastructure and policy planning to improve triage and transfer mechanisms. National Surgery Obstetric and Anaesthesia Plans (NSOAPS) have been developed in Africa with the aim of providing universal surgical care for all. Countries have adopted different NSOAP formulation models, such as the centralized model adopted by Zambia and Tanzania and the decentralized model adopted by Pakistan^21^. To date NSOAPS have not been adopted in Latin America^22^. Regional surgical plans following the NSOAP framework may be a route to improving subnational surgical care in regions that have strong national health care plans, but lack coordinated efforts focused at improving surgical care. This could be modeled similarly to the Pakistan approach where each province is developing a provincial surgical, obstetric and anesthesia plan (PSOAP)^23^.

### Limitations

The study is limited to the public sector of hospitals in the Amazonas, however we did not identify private hospitals in any municipalities surrounding the randomly selected hospitals surveyed. Due to our study design, we were unable to assess where there may be overlaps of catchment populations or situations where patients may bypass facilities to seek care directly in the capital. Due to logistical and financial limitations, two of the hospitals identified by randomization could not be assessed. While we implemented in depth facility assessments, many responses are based on administrative or chief surgeon input and are subject potential bias from these respondents.

## CONCLUSION

Although Brazil has a strong, federally funded national healthcare system, access to surgical care in remote areas remains limited. Surgical care delivery at rural, surgically capable hospitals does not reflect the surgical capacity in the state as a whole which relies on hospitals in the capital to provide the majority of surgical care. Increased investment in infrastructure for surgical care at these rural hospitals combined with the development of a state plan for the implementation of surgery and surgical triage is necessary to assure access to safe, timely, and affordable surgical care for the population of Amazonas outside of Manaus.
